# Building a healthy generation together: parents’ experiences and perceived meanings of a family-based program delivered in ethnically diverse neighborhoods in Sweden

**DOI:** 10.1186/s12939-024-02271-8

**Published:** 2024-09-11

**Authors:** Lisette Farias, Mai-Lis Hellenius, Gisela Nyberg, Susanne Andermo

**Affiliations:** 1 Department of Neurobiology, Care Sciences and Society, Division of Nursing, Karolinska Institutet, Huddinge, 141 83 Sweden; 2 Department of Neurobiology, Care Sciences and Society, Division of Occupational Therapy, Karolinska Institutet, Huddinge, 141 83 Sweden; 3 Department of Medicine, Karolinska Institutet, Solna, 171 77 Sweden; 4 The Swedish School of Sport and Health Sciences, Department of Physical Activity and Health, Lidingövägen 1, Stockholm, 114 33 Sweden; 5 Department of Global Public Health, Karolinska Institutet, Stockholm, 171 77 Sweden

**Keywords:** Disadvantaged populations, Family intervention, Health equity, Segregation, Migrants, Physical activity, Thematic analysis

## Abstract

**Introduction and aim:**

Ethnically diverse neighborhoods encounter pronounced inequalities, including housing segregation and limited access to safe outdoor spaces. Residents of these neighborhoods face challenges related to physical inactivity, including sedentary lifestyles and obesity in adults and children. One approach to tackling health inequalities is through family-based programs tailored specifically to these neighborhoods. This study aimed to investigate parents’ experiences and perceptions of the family-based Open Activities, a cost-free and drop-in program offered in ethnically diverse and low socioeconomic neighborhoods in Sweden.

**Methods:**

Researchers’ engagement in 15 sessions of the Open Activities family-based program during the spring of 2022, and individual interviews with 12 participants were held. Data were analyzed using reflexive thematic analysis.

**Results:**

The analysis resulted in three main themes and seven sub-themes representing different aspects of the program’s meaning to the participants as parents, their families, and communities. The main themes describe how parents feel valued by the program, which actively welcomes and accommodates families, regardless of cultural differences within these neighborhoods. The themes also show how cultural norms perceived as barriers to participation in physical activity can be overcome, especially by mothers who express a desire to break these norms and support girls’ physical activity. Additionally, the themes highlight the importance of parents fostering safety in the area and creating a positive social network for their children to help them resist criminal gang-related influences.

**Conclusions:**

The program’s activities allowed parents to connect with their children and other families in their community, and (re)discover physical activity by promoting a sense of community and safety. Implications for practice include developing culturally sensitive activities that are accessible to and take place in public spaces for ethnically diverse groups, including health coordinators that can facilitate communication between groups. To enhance the impact of this program, it is recommended that the public sector support the creation of cost-free and drop-in activities for families who are difficult to reach in order to increase their participation in physical activity, outreach, and safety initiatives.

## Introduction

Ethnic minority groups in high-income countries often belong to low socioeconomic groups, and have multiple long-term health conditions [1], higher rates of chronic diseases, and lower life expectancy [2]. Ethnic minority groups are heterogenous with variations in countries of origin, times of immigration, and resources. However, research shows that they face significant social and health inequalities, mainly related to conditions beyond their control [3], such as socioeconomic factors, lack of social support, stigma, and marginalization [4, 5]. These conditions can lead to unequal access to adequate housing, health services, physical activity (PA), and nutritious food [6]. In Sweden, 19.1% of the population of 10.2 million are foreign-born [7]. These foreign-born individuals encompass a diverse array of ethnic minority groups, often residing in suburban neighborhoods where significant proportions of residents have low educational attainment, low household income, high unemployment, and high disease and disability burdens [8, 9]. These neighborhoods are also often portrayed as problematic, ‘outside’ and excluded (*utanförskap*) from mainstream Swedish society [10]. The term ‘ethnically diverse neighborhoods’ is used here to refer to areas that are often socially segregated from their host society. These areas encounter diverse social challenges, yet they were intended to provide affordable housing for minority groups and low-income families with migrant backgrounds according to local standards [11].

Previous studies have shown that ethnic minority groups living in Europe are both less active and more sedentary than the general population [12]. A Swedish study found that the prevalence of obesity among immigrant groups living in socioeconomically deprived neighborhoods was up to three times higher than among the native population [13]. Further, research has shown that children from ethnic minority families are less physically active than their peers [14]. This is consistent with evidence indicating that childhood obesity is more prevalent among children from ethnic minority groups [12] and low-income families [13]. These studies emphasize the role that social status and economic resources play in maintaining optimal weight [14, 15].

Several factors can influence engagement in PA in ethnically diverse neighborhoods. For example, children from ethnic minority and low-income families often live in neighborhoods with limited access to safe spaces for play, which has been associated with lower levels of outdoor PA [16] and feelings of unsafety in their local environment [17, 18]. Concerns about personal safety have been identified as a barrier to use local recreational and PA facilities, for example, to play sports after school [12, 18], which promotes sedentary lifestyles [19, 20]. Although these barriers are multifaceted and complex, evidence has shown that whether individuals engage or not in PA and other health-related behaviors cannot be fully understood without considering the social context in which these activities take place [5].

Early health promotion programs that promote PA, such as outdoor play, are needed for children from diverse ethnic backgrounds. These programs should target children’s specific needs, preferences, and external constraints (e.g. economic and environmental inequalities) [21]. To promote children’s engagement in PA, health promotion programs should involve their parents. Parents can serve as an important mechanism of change for children’s health status by increasing their own healthy lifestyle behaviors [12] when provided with an environment that supports healthy behaviors [22]. Early health promotion efforts targeting families and children could benefit both ethnically diverse groups and society in general [23]. However, health promotion programs intended to support the general population, such as PA on prescription, may not be compatible with the situation of ethnic minority families with low socioeconomic status and living in deprived areas [24].

Previous research on adapting health promotion programs to groups living in disadvantaged neighborhoods emphasizes the importance of allowing time to build trust, preparing relevant material, training staff [25], and identifying and addressing cultural, religious, or contextual barriers to access and participation [26]. Identifying barriers to participation requires a more nuanced understanding of how health promotion programs are perceived and work in practice for families living in ethnically diverse and low socioeconomic neighborhoods. This understanding needs to consider the multiple dimensions of individuals’ lived experiences, including the different ways in which people interpret and participate in interventions and the contextual factors surrounding these experiences [25, 26]. One approach to gaining this understanding is to explore the experiences of those who participate in health promotion initiatives in order to learn from their preferences and needs [27]. Therefore, this qualitative study aimed to investigate parents’ experiences and perceptions of the family-based Open Activities program, as well as its meaning for them, their families, and communities living in ethnically diverse and low socioeconomic neighborhoods in Sweden.

## Methods

### Study design and research paradigm

To capture the complexity of promoting PA among families living in ethnically diverse neighborhoods, a qualitative research design was chosen [28]. This type of design allows for the exploration of participants’ subjective experiences, including the social context of their sense-making [29] when based on the research tradition of social constructionism [28]. Social constructionism facilitated researchers to better understanding of participants’ experiences concerning their specific socio-political, cultural, and historical contexts, and the perceived benefits of participating in the program as a result of their social interactions [30]. The study is reported according to SRQR guidelines [31]. The researchers involved in this study were all female and have backgrounds in designing, developing, and/or evaluating health promotion interventions for families, children, and disadvantaged communities.

### Study context

The Open Activities Program is a cost-free, drop-in, outdoor family program that welcomes families living in ethnically diverse and disadvantaged neighborhoods in Sweden. It was created by the Health Generation Foundation in response to the need for outdoor activities for families during COVID-19 in selected disadvantaged communities. The program consists of one hour of team-based activities in which parents and children are mixed together. The activities are led by a health coordinator once a week in a public park. The first 45 min consist of PA with an emphasis on promoting enjoyment of movement for both children and parents. The activities are intended for elementary and middle school age children, but everyone is welcome. The remainder of the 15-minute session is devoted to quizzes on healthy habits and the provision of fruit to all families. This program was chosen as the focus of this study since the research team has participated in several evaluations of this program and had access to the managers and health coordinators who lead the activities.

Since its launch in September 2021, the Open Activities program has been implemented in 16 municipalities in Sweden. However, in some municipalities, several ethnically diverse and low socioeconomic neighborhoods are being covered. Some of these areas are located in the suburbs of Sweden’s main cities, such as Stockholm, Gothenburg, Malmö, and Uppsala. One example of the areas covered by the Open Activities program is Rinkeby, situated in the northwest of Stockholm, where 92.1% of the population has diverse foreign backgrounds [32] The population in Rinkeby faces complex health challenges, including high rates of overweight, dental caries, and the highest exposure to daily smoking in the Stockholm region [33]. These challenges increase vulnerability to health inequalities in the area [34].

### Recruitment and participants

To minimize potential recruitment bias, all parents who participated in the Open Activities program with at least one of their children during Spring 2022 and Autumn 2023 were invited to participate in the interviews. This facilitated recruitment of participants of both genders and with different experiences (positive and negative) with the program [35]. Recruitment was conducted primarily through health coordinators during the program activities. Health coordinators provided written and oral information to all attending parents. After receiving the information, parents interested in being interviewed provided their contact information either directly to a health coordinator or to researchers who attended the activities. Researchers contacted all those interested via email or phone to schedule an interview at a time and place convenient to participants. By including all interested parents, selection bias was minimized as participants were not intentionally or inadvertently chosen to participate [35]. Twelve participants agreed to meet for an interview: nine mothers and three fathers with an average age of 43 years. The children of the participants involved in the Open Activities ranged in age from 9 to 12 years. Participants’ demographics are shown in Table 1.

**Table 1:** Participants’ demographics

Gender	Age	Civil Status	Number of children	Profession / Occupation
Female	43	Married	4	Preschool teacher assistant
Female	37	Married	4	Student Swedish as a second language (high school for adults)
Male	44	Married	3	Research Specialist
Female	48	Married	3	Preschool teacher assistant
Female	33	Married	2	Parental leave, work as a preschool teacher assistant
Female	48	Married	3	Preschool teacher assistant
Male	43	Married	2	Export operations worker
Female	33	Married	3	Part-time worker in-home care and student
Female	39	Married	3	Part-time worker as a doula and student
Female	49	Married	3	Student full-time
Female	48	Married	4	Assistant nurse
Male	48	Divorced	3	Warehouse worker

### Data collection

#### Researchers’ engagement

During the spring of 2022, researchers (LF and SA) were actively involved in the program. They participated in the Open Activities sessions for 1–1.5 h for 15 Saturdays to become familiar with the activities, the group of participants, and the work of the health coordinators. The researchers’ participation level was moderate to active [36] as they engaged in various activities and interacted with participants in different ways. For instance, they responded to humor, questions, and comments using verbal and non-verbal cues to demonstrate interest in the participants’ expressions during the activities. After each session, this engagement was documented, including the date, location, number of participants, physical environment, social interactions, type of activities, direct quotes from participants, and the researcher’s reflections.

#### Interviews

Interviews were conducted in either English or Swedish, at a time and place convenient for the participants. Out of the 12 participants, 11 preferred to conduct the interviews in Swedish, while only one requested using English. A semi-structured guide consisting of ten open-ended questions was used to prompt comprehensive responses. The interview guide was developed by the research team, drawing upon previous experiences of program evaluation and the research aim of this study. This was initially piloted with the assistance of a master’s student, who employed the interview guide in the presence of one of the researchers with few participants. Only minor modifications were made to the questions, primarily to simplify the language and facilitate comprehension for participants with limited proficiency in Swedish. Twelve interviews were conducted and recorded with parents, lasting from 25 to 46 min. Six participants requested to be interviewed immediately after the activities. One participant asked for the presence of a friend who could speak Swedish fluently because of language barriers. Considering time and work constraints, five participants agreed to be interviewed by cell phone, while one participant chose to complete the semi-structured interview guide by email to overcome language barriers.

### Data analysis

Braun and Clarke’s [37] six-phase process was used as a framework to conduct an inductive, reflexive thematic analysis (TA), which involved iterative movement back and forth through the phases. This interactive process and reflexivity were supported by researchers’ engagement in 15 sessions of the Open activities. Researchers’ participation in the activities allowed for a comprehensive understanding of the physical environment, social interactions, and the type of activities described by participants in the interviews. The researchers’ engagement in the activities led to the generation of more nuanced and contextual grounded interpretations of the data. These interpretations subsequently underwent further revision with the research team, contrasting them with the entire dataset and engaging in peer debriefing [38]. The entire data was systematically coded using the Atlas.ti 23 program, attending to all the data set with equal consideration. The program provided an accessible overview of the data and made it easier to back and forward across the transcripts.

The entire coded data was organized into preliminary themes, which were reviewed and refined by the research group. Some themes were combined and reorganized based on shared meanings. This involved generating themes and sub-themes by grouping the codes that shared a similar underlying concept or meaning across the dataset. These preliminary themes were checked against the coded extracts and the entire dataset. Several mappings of the codes were made using Atlas.ti 23 to facilitate visualization and reorganization of the codes and themes. The authors refined the themes through discussion, generating clear definitions and names for each theme. To ensure trustworthiness, the study’s quality was evaluated using Braun and Clare’s [29] questions for assessing TA. These questions were used to evaluate aspects such as the motivation for using TA, the type of TA employed (reflexive), and the alignment between the study’s epistemological underpinnings, the methods used for data collection, and TA.

### Ethical considerations

Informed consent and ethical approval were obtained before starting data collection. This study was approved by the Swedish Ethical Review Authority (Dr no: 2022-02643-02).

## Results

**Fig. 1:**
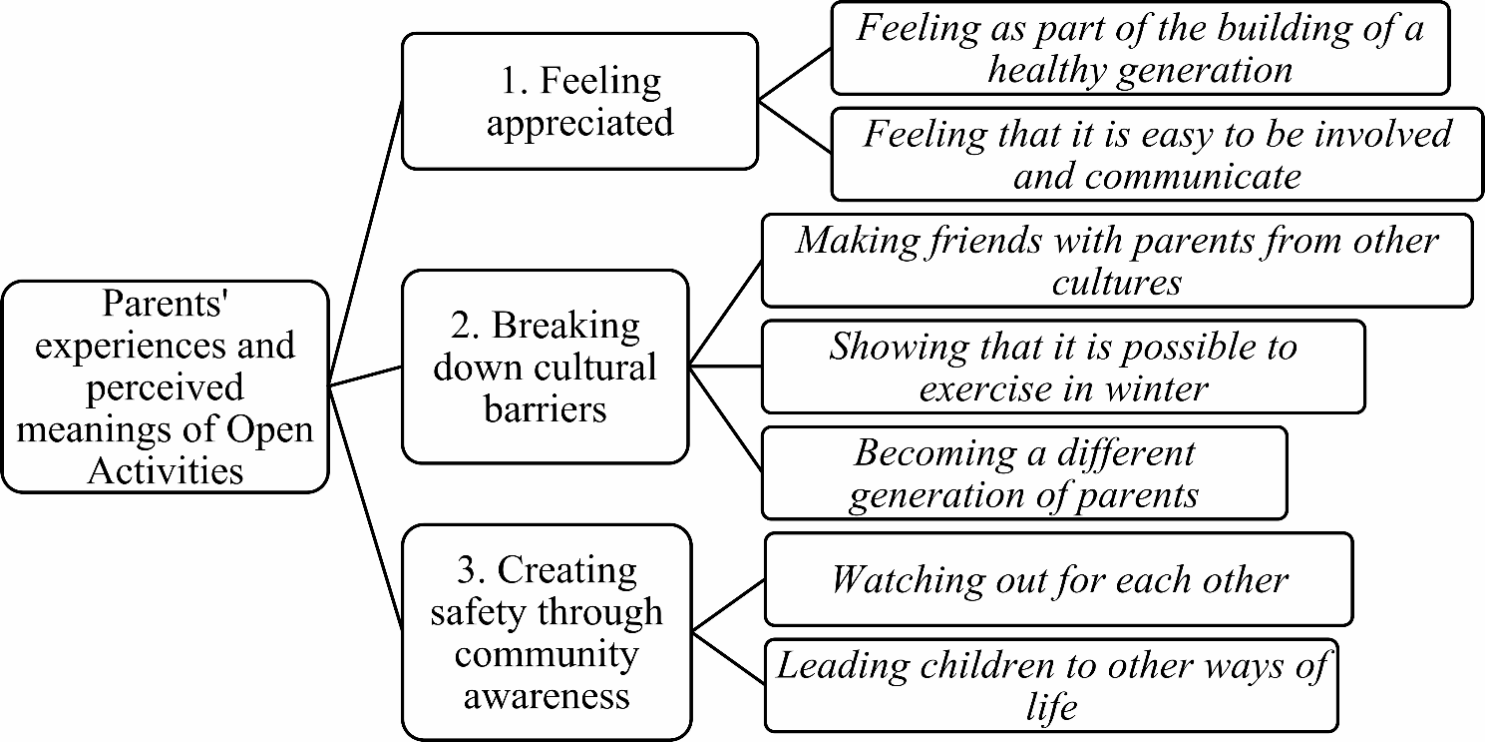
Themes and Subthemes

The three overarching themes reflect participants’ experiences and perceptions of the Open Activities program and its meaning for them as parents, their families, and their communities. Please see Fig. 1 for an overview of the themes and subthemes. The first theme acknowledges how families perceive the program as caring for them by working together to build a healthy generation of children and families in their neighborhoods. The second and third themes highlight the difficulties faced by families living in ethnically diverse communities where fear of going out and limited opportunities for PA are common. The second theme focuses specifically on the cultural values and norms that parents face when participating in PA, and how they overcome these cultural barriers by involving their families in the program. The third theme presents examples of how the program has helped families to create a sense of safety in the area by enabling parents and children to get to know and look out for each other outside of the activities. Additionally, the third theme illustrates how the program has provided children with a social circle that can help them resist pressure to engage in gang-related activities.

### Theme 1. Feeling appreciated as central

The first theme describes participants’ perceptions of their involvement in the program in terms of feeling valued as an important part of the formation of a new healthy generation in their communities. This appreciation is evident to the participants through the efforts made to make the activities easy to understand and follow, regardless of their level of Swedish.

#### Feeling as part of the building of a healthy generation

The first sub-theme is about how participants express that the availability of the Open Activities program in their community means that someone else cares about their situation, especially by involving parents in the activities. One participant expressed, “*I think it’s great to have the activities*,* but also to involve the parents*,* it is very important that they (the program) care about us too” (interviewee 8).* Parents described the program as a way to express appreciation for them, with particular consideration for those with multiple children and who face additional socioeconomic barriers to supporting their children’s participation in PA. This is illustrated by the following quote:

*It’s a kind of appreciation to us that I think it means something to the family*,* that they [the program] appreciate us*,* We*,* families that have many children*,* there will be 3 or 4 children from one family there. That’s something. That attention to this type of family. (interviewee 6).*

This sense of feeling appreciated by the program was important as parents often reported that “*the most important thing in this area is feeling welcome*” (interviewee 5). Additionally, the parents expressed the importance of feeling that they were contributing to the building of a healthy generation of children and families when they met through the Open Activities program. In this sense, being part of the Open activities provided families with a more meaningful everyday experience that was distinct from their daily routine consisting of going to work, school, and home. For instance, one parent explained:

*We build a healthy generation when we meet. We meet each other*,* have children’s activities*,* and have (good) communication*,* they (the program) appreciate us*,* and we appreciate them too. It’s not just going to school*,* pre-school*,* and back from school*,* pre-school*,* there’s also a separate thing in our lives that is? free*,* that is also important (interviewee 6).*

*You work full-time and especially in areas like this*,* it is very tough*,* and then you get home you also have to cook and to pick up your children from school and so on*,* but on the weekend*,* then you have a time for yourself to have an activity ( Interviewee 5).*

#### Feeling that it is easy to be involved and communicate

The participants described the importance of having activities that were easy to understand and that were inclusive of all people, including those who were not fluent in Swedish. This was especially important because their communities welcome families from diverse backgrounds, and it was crucial to integrate children who do not speak the language. A parent emphasized the importance of welcoming non-Swedish-speaking children:

*Some children do not know Swedish*,* which we also have to consider very much in this area*,* we should take them in*,* at least as I understand it*,* you don’t not need to know all the language just to play a game*,* they should learn (Swedish) this way (interviewee 4)*.

Although the activities support non-verbal communication and inclusion of those who do not speak Swedish, some participants expressed alternative perspectives. They noted that many parents were apprehensive about participating in the activities due to their limited proficiency in Swedish. For instance, parents were concerned about making mistakes or saying something inappropriate that could lead to problems with social services. They shared examples where other parents had been told that individuals could interact with their children in ways that might be perceived as problematic “*if you say that your mummy hits you*,* then you will get this”*. One participant said that:

*I have a friend for example who doesn’t know the language very well and she has had problems with social services*,* so she doesn’t want to take children to the activities*,* so I say to her ‘I know exactly how to talk to your children*,* and I know exactly what to say if they say something wrong*,* how to discuss it with this person (health coordinator)’ (Interviewee 5)*.

Participants reflected on the benefits of taking part in the program when considering the ethnic diversity of their communities. They noted that having a leader, such as a health coordinator, made it easier for individuals to follow instructions and just have fun without having to worry about knowing the rules of the activities. For instance, a participant explained *“there is a leader (health coordinator) who explains using body language*,* so the activities have so many great benefits because there is also this energy. I don’t know how to explain this energy*,* but no one gets worried” (Interviewee 10)*. Another parent described it as *“I don’t have to explain to anyone because a leader (health coordinator) is there*,* and he/she explains to everyone how to play. And then I just have fun with my kids. You can’t put a price on it*,* it’s so much value (Interviewee 12)”.*

Other parents also acknowledged the importance of having a leader (health coordinator) to support communication between families, regardless of cultural or language differences.

*There are a lot of immigrant groups coming into these areas and when you come in and you don’t know the language and you don’t know it as an adult then you don’t know the culture. If there is a leader*,* a game where the children can be involved and can come in*,* so it’s easier to communicate with each other if they know English*,* so you can communicate and help each other*,* it becomes more open. You become more like everyone since everyone does the same thing (Interviewee 10)*.

### Theme 2. Breaking down cultural barriers

The second theme highlights how the program supported parents in changing their cultural beliefs to support their children’s engagement in PA. It also illustrates that cultural differences exist among ethnically diverse groups living within the same area, as well as cultural differences between different generations of the same family.

#### Making friends with families from other cultures

Participants described how they found that they could have fun with other families through the activities, regardless of differences in background, religion, and cultural traditions: “*it doesn’t matter what identity you have or religion or skin color or where you come from*,* that we are all together and have fun. It’s wonderful” (interviewee 8).* Participants gave examples of how social media and other sources can sometimes make it difficult to build relationships between ethnically different groups because these sources focus more on their differences. They described how the activities facilitated a first contact between families with diverse backgrounds and how their children were able to see beyond differences and even start to contact other families outside the program.

*You get to meet other people and also that everyone has different religions*,* I think that’s great because of social media*,* it has become very much like this ‘you are Jewish*,* you are Muslim*,* you are Christian*,* you are fasting’*,* so I feel that when children are involved in these activities*,* you forget what religion you come from*,* they forget and then you learn to be with other people and we parents can also meet other parents and then start to find activities together in addition to the program (interviewee 5)*.

Participants also highlighted how their children were key in helping them to think more openly about other groups. They realized that they needed to make an effort to get to know other families as they wanted their children to make friends through the program.

*The children exchange phone numbers and socialize with each other and that’s it. They don’t give a damn about anything that has to do with culture or anything like that*,* because they don’t see it in the same way*,* so as a parent you have to make that contact. You want the children to have friends(interviewee 10)*Conversely, some parents found it challenging to try to get to know other families. Initially, there was a need to identify which families were inviting their child to play, and address concerns about entrusting their child with unfamiliar individuals or visiting the homes of people they did not know.

*I didn’t know them then*,* but my child decided she wanted to go with this child. Okay*,* who is the parent? We are very careful now; it is not like our time when we were little. I don’t know them. How would I let it get home to them? I had to make an effort and at least talk to them [with the parents] (Interviewee 8).*

Realizing that all the families involved in the program wanted the best for their children was the first step for the parents in breaking down prejudices and other barriers between different ethnic groups in their communities.

*We have come close to other families and many prejudices*,* and that rigidity and coldness have died after some time*,* so we have started to greet each other when we are outside the activities because we live close. So*,* it has been good*,* on all some families have got to know each other and begun to be respected (interviewee 12)*.

#### Showing that it is possible to exercise in winter

Other cultural barriers perceived by participants as reframing their experience of the program were their beliefs about exercising or going outside in cold weather. For example, some participants recognized that they would not let children play outside during winter.

Other parents would not go for long walks or exercise in winter because they had not been exposed to these experiences before. *“if it is cold*,* it is impossible for the children to move” (Interviewee 1).*

*I usually don’t wait for buses or trains for more than 5 min*,* instead*,* I like to walk to the next station*,* that’s my principle*,* but even if I don’t dare to do it in the cold*,* in winter*,* I still see some people running and doing some exercises*,* it could be that we don’t know how to do the exercise in winter*,* as we are like people who come from different kind of lifestyles (interviewee 3).*

This lack of experience has led some families to prevent their children from trying winter sports. One participant shared: “*I don’t know them [sports]*,* so I don’t encourage my children to do them. I can give you the best example*,* ice skating*,* so I couldn’t understand myself because I wasn’t exposed to it in early childhood”(Interviewee 3).* Other parents realized through their participation in the program that their beliefs about the weather were linked to their cultural backgrounds and that this prevented some families and children from participating in the program during the winter.

*I think there are many where we live*,* that have this little ‘it’s too cold’ so ‘how can we have our kids outside*,* what if they get sick’. There’s a lot about that*,* it’s in the middle of this culture. You think ‘if you’re outside too much you get sick’. Culture is very much like this*,* ‘if you go out without a jacket. Then you will get sick’. ‘Put on gloves*,* otherwise*,* you’ll get sick’. We believe that everything makes you sick and that’s perhaps the reason why people don’t go to activities as much in the winter because it’s too cold (interviewee 8).*

After taking part in the program, some parents realized that they could have fun even when it was raining or cold outside and that they could pass on this attitude to their children: “*It rained but the activity was great fun*,* then I think that when the children come*,* we should communicate with them talk to them”(interviewee 4).* Another parent gave a similar example, including the realization that trying new winter activities through the program made her appreciate this opportunity and learn from this new experience.

*It is great fun with different activities that I would not have had the chance to try if I had not been involved in this program […] I’m grateful and before you’ve tried*,* you don’t know that it’s even such a feeling to be able to do it. You might think no*,* I can’t handle this*,* or this doesn’t suit me. Or what’s the point? But once you have tried it*,* it feels great that you still have that experience right now (interviewee 9).*

#### Becoming a different generation of parents

This next sub-theme illustrates some of the ways in which parents have actively tried to overcome cultural beliefs that have prevented their own or their children’s participation in PA. When one participant spoke about her parents’ cultural beliefs, she said, ‘*I played football and danced*,* but because of our culture I wasn’t allowed to continue. In our home countries it was not a good reputation*,* so my father was very old-fashioned’(interviewee 5)*. This background motivated this parent to involve her family in the program and become a new generation of parents, breaking previous cultural barriers, ‘*we ‘the new’ generation of parents have grown up here*,* so we have learned how society is and we don’t want our children to be like we were*,* like our parents were at that time’ (interviewee 5).*

Other mothers also described the need to break down cultural barriers to become a different generation of mothers who could play with their children in the program. One participant shared that when mothers are doing the cooking, shopping, and cleaning, they do not have time to play with their children who are often in front of the computer or TV. But when they started to be active in the program’s activities, they realized that this was fun, even though their participation was not always well received or understood in their communities.

*When we go to the activities and share time with our children*,* this is the most important time for the children and it’s also fun for me. When I was little*,* I liked such [games]*,* so it’s also fun when our parents are involved and we laugh*,* maybe they also look at us. They laugh at us*,* because we adults are also there*,* you know this area. They think mum can’t do what the kids do (interviewee 6)*.

For some mothers, however, participation in the activities proved challenging. One father elucidates the difficulties his female partner encountered and expresses a desire to change her perspective, which he believes is shaped by traditional cultural norms that are at odds with the program’s expectations.

*Women are not ‘outside kind of people’*,* they are considered more in the house and close to our home*,* so if not related to some agricultural activities they can’t go out and then exercise*,* go running or something like that*,* it [the program] is very nice compared to my experience and background*,* this is an ideal scenario (Interviewee 3)*.

Overall, participants became aware of the ways in which their parents’ beliefs and cultural backgrounds had hindered their participation in PA. They also recognized how the program provides them with an opportunity to become active themselves and support their children’s participation in PA, regardless of how their communities might view their involvement. By being active, they realized that their children saw them differently and allowed them to create meaningful parent-child relationships.

*I think it’s good that parents are involved in the activities so the children see us in a different way*,* they think that we are just going to stand there and stare and kind of be a guard for them*,* but when*,* for example*,* my children see that I play in activities and last time I fell in the goal area*,* then they think it’s fun and they said " you played with us and it was great fun to get adults to play with us” so it’s clear that they see it in a different way (interviewee 5)*.

*Mothers should not only cook and go to the store and come to work. They can play with their children also*,* you don’t have to have children sitting at the computer*,* when we go there [to the activities] we share time with our child*,* this is the most important time for the children (Interviewee 6)*.

### Theme 3. Creating safety through community awareness

The third theme captures the importance of providing the Open Activities program in ethnically diverse neighborhoods, which in turn allows them to build positive connections for their children, awareness, and a sense of safety within the community.

#### Watching out for each other

Participants acknowledged that the program contributes to a sense of safety within the community as families get to know each other better across ethnic groups. Recognizing participants on the street has given participants the feeling that “*you’re not afraid of anyone” (interviewee 12)* because they perceive that families involved in the program have good intentions. “*There are more people that come out*,* so the more adults there are[outside]*,* then it becomes better*” (Interviewee 10).

*It can contribute to a little more safety in the neighborhood where you live because you know each other. You know that no harm will come from that person because you have a joint activity. I think it’s great for the children*,* too*,* when they see someone*,* they know out there*,* they feel a little more secure (interviewee 9)*.

One example provided by participants suggests that they have become more aware of each other and have started to look out for each other’s children when they meet them on the street.

*If we see a family outside the activity*,* if we see them alone on the street*,* we are more aware of the children that we see in the program*,* so we are a bit more attentive. So that you don’t have a situation that you don’t want to happen to your children*,* it’s safer. The families also do it for other children. So*,* you are not afraid of anyone or anything (interviewee 12)*.

An alternative perspective was provided by one parent, who did not describe the activities in terms of creating safety, given that he had no concerns about the area since he felt that he had no option but to remain in the area.

*I am not exactly worried*,* I heard from the news and from people or some friends ‘oh this has happened*,* this and that has happened in your area’*,* but I keep working*,* I don’t know if there is a choice to move*,* to be honest*,* there is no choice and I’m not scared of living here*,* but it’s not my choice (interviewee 3)*.

#### Leading children to other ways of life

With her own children in mind, one participant expressed her fear that children and young people in her neighborhood might meet the ‘wrong’ people and become members of gangs who are looking for young people to do small errands. She describes the program as giving her children positive role models and a network that could help avoid the ‘wrong circles’.

*I just think it’s good that there is someone who really likes to put energy into such activities*,* especially in vulnerable areas. So we need them*,* many parents don’t have the time to take their children out*,* many parents can’t afford to put their children in activities and so on*,* and right now it’s very worrying because all those who are between 14 and 17 years old are the ones who are the ‘errand boys*^1^*’ in the areas*,* so I think it’s good that there’s someone who can lead them to another path. My son could end up in the wrong circle*,* so I think it’s good that you have the activities*,* I like living in the area and then I have to fight a little extra so that my children don’t end up in the wrong circles (interviewee 5)*

Thinking about the future, one participant also emphasizes the importance of having her children in the program. Getting to know other children could protect them in the future, as others will know who they are, which could prevent any possible misunderstandings that could get their children into trouble.

*Even if you don’t talk to each other*,* you know each other*,* and the children realize that when they get older*,* they know that everyone knows who they are and that makes a lot of difference (interviewee 10)*.

*You become a little more responsive. So*,* it doesn’t happen a situation*,* something that you don’t want to happen to your children. It’s safe. The families keep up with other families (interviewee 12)*.

## Discussion

The present study investigated parents’ experiences and perceptions of the family-based Open Activities program and its meaning for them, their families, and communities located in ethnically diverse and low socioeconomic neighborhoods in Sweden. The findings indicate that participation in the program allowed participants to break down cultural and gender barriers that had previously hindered their involvement in PA. Additionally, it was perceived as fostering social cohesion, sense of safety, and positive social networks for the children participating in the program.

### Family-based PA interventions with ethnically diverse groups

A systematic review of family-based PA interventions indicates that the delivery of activities by culturally appropriate leaders is a crucial aspect of engaging children from ethnic minorities in PA [39]. Nevertheless, it is not always feasible to match staff to participants’ diverse ethnic backgrounds. For instance, the Open Activities program attempts to recruit health coordinators/leaders from the same areas as the participants. However, the diversity of cultural backgrounds in these areas may restrict the leaders’ ability to match all cultural preferences. Previous studies on family-based interventions indicate that a potential solution to this issue is to develop effective communication strategies that prioritize sensitivity to language use and information requirements [26]. This approach is supported by findings from scoping reviews of similar family-based PA programs, which recommend that leaders focus on facilitating communication in non-English speaking communities by using language adaptations [25, 40]. As illustrated in the present study, health coordinators used nonverbal communication, English language, and peer support to facilitate communication. These strategies were used to include those who were hesitant to speak due to lack of vocabulary or confidence, and fears of being misunderstood. It is therefore proposed that facilitating communication may serve to enhance participants’ feelings of welcome and perception of leaders as caring and committed to integrating them into the activities.

### Heterogeneity and gender barriers to PA participation

The findings show that families living in ethnically diverse neighborhoods may have different cultural values, which can pose challenges when trying to connect with other families in their area. This is an important finding because the perception of families as homogeneous with shared cultural norms is unfair and can be misleading [41]. These cultural values can also create conflict among families, potentially hindering the development of a sense of community [42]. Therefore, it is worth noticing that the Open Activity program served as a neutral space to focus on families’ health rather than existing cultural differences.

Similar to the findings of a scoping review exploring barriers, enablers, and cultural adaptations of PA interventions for minority ethnic groups [43], this study has shown that some gender norms can become a barrier to PA. In the scoping review [43], expectations for women such as to prioritize family and domestic responsibilities, limited their time available to engage in structured PA. This is consistent with the results of a study of women from diverse ethnic backgrounds in Sweden, which indicated that their communities may perceive participation in PA as incompatible with the roles of married women/mothers [38]. In line with these findings, the Open Activities program afforded mothers the opportunity to engage in PA with their children, thereby avoiding any neglect of their caregiving duties while simultaneously facilitating a shift in those responsibilities. This resulted in a more positive relationship with their children, who were able to observe their mothers assuming roles outside the home. However, a potential limitation of the program is that the majority of mothers participated with their children alone, which may have perpetuated a gendered perspective of mothers as the primary caregivers for their children.

Previous studies also shown gender restrictions on girls’ PA participation [17, 44]. Some older generations of parents from multi-ethnic backgrounds may view certain activities, such as football, as a threat to cultural traditions [17]. However, this study shows that new generation of parents (younger or born in the host country) may be particularly supportive of their daughters’ engagement in PA [45, 46]. For instance, mothers who had previously been denied the opportunity to participate in football by their parents felt that the Open Activities program helped them break away from these norms to encourage their daughters to participate in PA. Another factor described as a major barrier to PA in studies focusing on ethnic minority groups is exercising in bad/cold weather [17, 47, 48] as a causal mechanism to explain illness [49]. These findings are somewhat contradictory to those of the present study. Women in this study indicated a belief in the causal relationship between cold temperatures and illness. However, they also expressed a willingness to go out during the winter months if the activities were held indoors. Consequently, a potential limitation of the Open Activities program is the lack of emphasis on raising awareness of the benefits of PA to challenge the beliefs that link cold weather and illnesses.

Other gender-barriers to PA described in qualitative studies with ethnic minority groups are self-identifying as non-exercisers and lacking experience with exercise in their home country [50, 51]. Although some of the women in this study could related to the barriers described above, they perceived the program as an opportunity to (re)discover PA with their children, even though their communities may disapprove of their participation.

### Neighborhood perceived safety and PA participation

Consistent with previous qualitative studies on barriers to PA in ethnically diverse neighborhoods, the findings of this study illustrate that the perception of neighborhood safety is a key factor for families to engage in PA [27, 52, 53]. Although some neighborhoods may have good access to outdoor spaces, safety concerns can hinder participation in outdoor PA [54]. Expanding on this literature, participants in this study emphasized the importance of the Open Activities program as a safe place to meet other families, as well as having health coordinators and other adults promote safety and positive role modeling. This insight is in line with research showing that the presence of safe adults is associated with more frequent PA in these neighborhoods [53].

The potential long-term impacts of the Open Activity Program on community cohesion and health outcomes include the fostering of positive and healthier networks and pathways for the children participating in the program. For example, children who participated in the program may have gained new experiences with PA that could facilitate continued engagement in PA as they mature. While financial and time constraints may persist over the long term, these children may find support in other children and families involved in the program to identify cost-free activities that will enable them to remain active. Other potential impacts pertain to the networks fostered by the program, which may facilitate mutual support among families and contribute to the prevention of gang recruitment or misunderstandings with law enforcement or social service agencies. In line with prior research [52], participants in this study indicated that establishing connections with other adults could offer their children role models and alternative life pathways, thereby preventing involvement in criminal activities.

### Strengths and limitations

The relatively small number of participants may be viewed as a potential limitation. However, it should be noted that recruiting ethnically diverse groups residing in segregated neighborhoods has proven to be a significant challenge [55]. Nevertheless, the accounts provided by the participants yielded invaluable insights that can inform the development of culturally sensitive PA family-based programs for other communities facing similar challenges. It is noteworthy that there were few, if any, cost-free programs available to participants in their areas, and that they encountered significant economic and time-related challenges in engaging their children in PA. This could explain their positive views about the program. Another potential explanation is that parents who were dissatisfied with the program had already discontinued their participation by the time the researchers joined the activities, resulting in a limited number of alternative or discrepant views in the analysis. To facilitate critical reflexivity [56], note taking and peer debriefing were used by the researchers to discuss their impressions of the context and participants’ engagement with the program.

### Implications

Practice implications include developing culturally sensitive activities that are cost-free and take place in local public spaces to enhance accessibility. It may be beneficial to tailor activities and recruit leaders who possess a high degree of cultural sensitivity to enhance participation in PA of groups that are less active. An understanding of the cultural sensitivities that shape social and gender norms is crucial for identifying factors that may either encourage or discourage participation in PA. As an alternative to the Open Activities program, it is recommended that the development of gender-specific activities (e.g., only women or girls’ activities) be considered for communities who may have cultural or religious values that impede women’s participation in PA. It is crucial for policymakers to acknowledge the interconnection between safety concerns and sedentary and unhealthy lifestyles. Similarly, it is recommended that contextual barriers impeding access to public spaces, such as parks, be addressed by allocating resources to enhance their visibility, lighting, and proximity to public transit. By enhancing the safety of public parks and providing support for culturally sensitive, cost-free physical activity initiatives, it may be possible to increase the engagement in PA of hard-to-reach populations, such as ethnically diverse groups.

## Conclusion

This study contributes to the development of family-based PA programs with populations that have proven challenging to reach, such as ethnic minority groups. The study demonstrates the potential of such programs to serve as a neutral space for parents to connect with their children and other families, foster a sense of community, and (re)discover PA. This (re)discovering was shown to be especially important for women and younger generations of parents who found an opportunity in the program to challenge gender norms and support the participation in PA of their daughters. Further studies could examine the advantages and disadvantages of adapting family-based PA programs to align with specific cultural or religious values. One potential approach would be to utilize sessions exclusively for women or girls, with the same-sex health coordinators. Given the predominance of mothers in this study, future studies could investigate the experiences of fathers in family-based programs and the challenges associated with their recruitment and engagement in PA.

## Data Availability

No datasets were generated or analysed during the current study.

## Footnotes

1 An errand boy (springpojkar in Swedish) is usually a young boy with low socioeconomic background who runs small errands to gangs or criminal groups like selling drugs for little money.
